# Effect of a Three-Hour Written Examination on the Nerve Conduction Velocity (NCV) of the Dominant Hand in Medical Students

**DOI:** 10.7759/cureus.98030

**Published:** 2025-11-28

**Authors:** Manju Chenicherry, Sunaina Soni, Jitender Sharma, Anjum Datta, Isha Soam

**Affiliations:** 1 Physiology, Andaman and Nicobar Islands Institute of Medical Sciences, Port Blair, IND; 2 Physiology, All India Institute of Medical Sciences Central Armed Police Forces Institute of Medical Sciences, Maidangarhi, Delhi, IND; 3 Neurology, Army College of Medical Sciences, New Delhi, IND; 4 Physiology, Subharti Medical College, Meerut, IND; 5 Physiology, Army College of Medical Sciences, New Delhi, IND

**Keywords:** dominant hand, median nerve, medical students, nerve conduction studies (ncs), nerve conduction velocity (ncv), objective examination, subjective examination

## Abstract

Nerve conduction studies (NCS) help diagnose and monitor disorders of the peripheral nervous system. The nerve conduction velocity (NCV) test is a simple, non-invasive method for evaluating nerve injuries and neuropathies. Various factors, including age, temperature, and limb dominance, influence NCV. Fatigue, which can be physical or mental, also affects nerve conduction. Continuous activities like writing can lead to muscle fatigue, but motor redundancy helps compensate for reduced function. Hand dominance, assessed using the Edinburgh Handedness Inventory (EHI), may affect NCV. This study examined NCV changes in the dominant hand of medical students after three-hour objective and subjective exams. The study involved 50 first-year medical undergraduates who consented to participate, and was conducted in the Physiology and Neurology departments of a reputed medical college in North India. We hypothesized that the objective examination would cause less fatigue to the muscles of the hand, hence it could assist in bringing a change in the pattern of university examinations. To the best of our knowledge, this study is the first of its kind, as no study has previously assessed post-examination NCV in medical students. However, the results of the study showed little change in the conduction velocity of the motor part of the median nerve. This could have been due to the small sample size.

## Introduction

The nerve is like an electrical wire through which electricity/impulse passes from one point to another. Motor activity of a muscle occurs when the impulse is transferred from the nerve to the muscle. If the nerve is damaged, this transmission is disrupted, leading to impaired motor function in the affected muscle. Nerve conduction studies (NCS) are performed for the diagnosis and prognosis of disorders of the peripheral nervous system. One of the key components of NCS is the nerve conduction velocity (NCV) test, which is a simple, non-invasive method used for diagnosis, localization, monitoring, and prognosis of nerve injuries and neuropathies. This test is also done as part of a comprehensive evaluation of muscle and nerve function. Several factors can influence NCV results, including age, temperature, climate, fatigue, stress, and whether the nerve tested is in the upper or lower limb. In cold conditions, the limbs may need to be warmed to ensure accurate measurements. Prolonged activities such as continuous writing can lead to fatigue in the intrinsic hand muscles, potentially affecting test results [1]. A reduction in the conduction velocities of peripheral nerves was found in computer operators who worked for long hours, along with symptoms of pain, paresthesia, and weakness [2].

The Edinburgh Handedness Inventory (EHI) [3] is a questionnaire to assess an individual's handedness, i.e., their preference for using one hand over the other for various tasks. It includes items that ask respondents to indicate their preferred hand for activities such as writing, throwing a ball, and using scissors. In the present study, the EHI was employed to determine hand dominance among participants. Although numerous studies have examined the relationship between hand dominance and NCV [4-6], this study is novel in that it investigated the NCV of the dominant hand in medical students, after subjective and objective examinations in the same setting. A NCS involves stimulating nerves at multiple sites, typically in the limbs, using small electrical impulses and recording the resulting responses. Surface electrodes are utilized for both stimulation and detection. Prior research has demonstrated that NCS is a safe procedure [1,7].

Since school, students have been molded to conform to a particular examination pattern. Most of us are trained to memorize specific topics and reproduce them in three-hour-long subjective examinations. This approach to assessments and curriculum design has, perhaps unintentionally, encouraged rote learning across various levels of education, from school to professional courses. Even teaching practices are such that they tend to prioritize memorization over genuine understanding of the subject matter [8,9].

The current examination system in medical colleges is the same as that in schools, relying heavily on long, subjective assessments. However, there is a growing need to shift towards a system that prioritizes critical thinking, problem-solving abilities, and real-world application over rote memorization [9]. An alternative approach to addressing this issue is the introduction of objective examinations within the curriculum, as these are known to foster analytical thinking in students. The present study aimed to investigate how subjective exams may potentially affect students' physical well-being, specifically muscle strength. We assessed differences in NCVs following a three-hour subjective versus objective examination, reflecting the typical duration of university exams in Indian medical colleges. To the best of our knowledge, this research question has not been explored previously. Therefore, we hypothesize that prolonged subjective examinations would lead to a measurable decrease in NCV due to fatigue in the dominant hand, and could serve as evidence for linking exam format to physiological stress. In the future, this would pave the way for a potential shift in examination pattern in the medical education system.

## Materials and methods

The present study was a pre-post test comparison study involving first-year medical students from the Army College of Medical Sciences, Delhi Cantt., a reputed medical college in North India. Ethical clearance for the research was obtained from the Institutional Ethics Committee of the Base Hospital & Army College of Medical Sciences, Delhi Cantt. (IEC/02/2023/40). The sample size was calculated using the appropriate statistical formula:

N=[(Z_1−α/2_​×SD)/E​]^2^

Where z_1 - α/2 _=1.96 for a 95% confidence level, SD=3.9m/s, E=1.5m/s. The sample size was adjusted to 50 students who consented to participate in the study. The data for this study were collected from the same group of students under three different scenarios. Participants included healthy medical students, as confirmed by medical records at the time of admission to the medical college, and were between 18 and 23 years of age. Students with autoimmune diseases, autonomic dysfunction, thyroid disorders, psychiatric illness, on medication, with a pacemaker, implanted stimulator (cardiac implants, deep brain stimulators, spinal cord stimulators), and neuromuscular deformities of the upper limb, and differently abled ones were excluded from the study.

Study design and procedure

The experimental procedure was conducted over three consecutive days to assess NCV under three distinct conditions:

Day 1 - Baseline Measurement

Baseline NCV values were recorded on the first day. Measurements were taken after confirming that the participants were in a rested state, free from any recent academic engagements, examinations, or other external stress-inducing factors. All participants were seated comfortably during the procedure, which was carried out in a calm and quiet environment in the Neurology Laboratory of the affiliated hospital.

Day 2 - Post-objective Examination Measurement

On the second day, participants completed an objective examination on a pre-specified topic. The topic was chosen to be consistent across all participants to ensure academic uniformity and minimize variability in emotional or cognitive stress levels due to differing perceptions of difficulty. This assessment consisted of multiple-choice questions (MCQs), designed to simulate a more analytical, time-constrained, and performance-driven form of academic evaluation. The exam was paper-based with 200 single-response type questions, and the time duration was three hours. Students were asked to write answers a, b, c, or d in the boxes provided in the question paper. NCV was measured immediately after the examination, allowing for the capture of examination-related psychological and physical stress. 

Day 3 - Post-subjective Examination Measurement

On the third day, participants underwent a subjective (essay-type or theory-based) academic examination based on the same topic covered in the previous day’s test. The subjective examination included three long essay-type questions and 10 short-answer questions. NCV was measured immediately after the examination to capture physiological changes associated with this type of examination.

All tests were conducted using a standardized procedure under the supervision and technical support of experienced personnel from the Department of Neurology. The testing apparatus included a multi-channel nerve conduction system, calibrated for both motor and sensory conduction studies. For consistency across participants, the dominant hand (most commonly the right hand) was used for all measurements. Motor nerve conduction velocity (MNCV) was recorded from the median nerve.

The room temperature was maintained uniformly across all sessions (22-24°C), and participants' skin temperature was kept between 32 °C and 34°C, as temperature fluctuations are known to affect NCVs. All tests were conducted during the morning hours (between 9 AM and noon) to minimize diurnal variation in physiological parameters. Participants were instructed to remove any topical lotions from their upper limbs and refrain from consuming caffeinated drinks, alcohol, drugs, smoking, or engaging in any physical exercise before testing.

Statistical analysis

Statistical analysis was carried out using IBM SPSS Statistics for Windows, Version 25 (Released 2017; IBM Corp., Armonk, New York, United States). Data were examined for the assumptions of normality using the Shapiro‐Wilk test statistic and homogeneity of variances using Levene's test. The mean conduction velocity was compared between the conditions using analysis of variance (ANOVA) and between the genders using an unpaired sample t-test. A p-value of <0.05 was considered to report statistically significant findings.

## Results

The mean (SD) age of the subjects was 19.86 (1.07) years. The mean NCV of the motor part of the median nerve in meters per second (m/s) at baseline, post-objective, and post-subjective exam are shown in Table 1, with no significant difference between the three conditions (p=0.646).

**Table 1:** Motor nerve conduction velocity (NCV) of the median nerve in subjects (N=50) between conditions

	NCV (m/s)		P value (ANOVA)
	Mean	Standard deviation	
Baseline	65.400	7.09	0.646
Objective	64.198	6.49	
Subjective	64.458	6.68	

The NCV (m/s) was significantly higher in female participants compared to male participants at baseline, post-objective examination, and post-subjective examination (p<0.01) (Table 2).

**Table 2:** Motor nerve conduction velocity (NCV) of the median nerve between female and male subjects in different conditions

Study group (category division)	Gender	NCV (m/s)		P value (independent t-test)
		Mean	Standard deviation	
Baseline	Female	69.000	7.3567	0.0001
	Male	61.800	4.6387	
Objective	Female	67.012	6.6407	0.001
	Male	61.384	5.0537	
Subjective	Female	67.960	6.4558	0.0001
	Male	60.956	4.8843	

However, no significant differences in NCV were observed between the three conditions.

There was no significant difference in NCV (m/s) between baseline, post-objective, and post-subjective exam in female and male subjects (Table 3).

**Table 3:** Motor nerve conduction velocity of the median nerve between conditions in the female and male subjects

Gender	Study group	NCV (m/s)		P value (ANOVA)
		Mean	Standard deviation	
Female	Baseline	69.000	7.3567	0.591
	Objective	67.012	6.6407	
	Subjective	67.960	6.4558	
Male	Baseline	61.800	4.6387	0.829
	Objective	61.384	5.0537	
	Subjective	60.956	4.8843	

## Discussion

The present study aimed to assess differences in the NCV of the motor part of the median nerve in the dominant hand following three hours of subjective and objective examinations in first-year medical students, and review the current examination system in India, which may unintentionally encourage rote learning among students. Although demographic variables were comparable across groups, female participants exhibited significantly higher NCV than males at baseline, post-objective, and post-subjective assessments. However, the primary finding of this study is that there was no significant difference in NCV between the three conditions, i.e., baseline, post-objective, and post-subjective examinations.

Prolonged stress during subjective examinations may impact upper limb function through mechanisms such as nerve compression, reduced blood flow, and muscle fatigue, as supported by existing literature [10]. Previous studies have demonstrated significant prolongation of latencies, reduced amplitudes, and slower conduction velocities with increasing age compared to younger individuals [10]. Additionally, female subjects have been reported to exhibit shorter distal motor latencies (DMLs) and F-wave latencies in the median, ulnar, and peroneal nerves, whereas male subjects tend to show lower sensory amplitudes in the upper extremity nerves [1]. Although the present study did not assess the effect of handedness on NCV, previous research suggests that MNCV tends to be higher in right-handed individuals, while sensory NCV is greater in left-handed individuals compared to their counterparts [11,12].

NCV has been widely used as an assessment tool in conditions such as hand-arm vibration syndrome (HAVS), carpal tunnel syndrome (CTS), and various sports-related injuries [13-16]. To the best of our knowledge, no previous study has investigated the effect of continuous written examinations on NCV. Although the current study did not reveal significant differences across conditions, future research involving a larger sample size may help elucidate potential changes in NCV induced by prolonged written examinations.

The limitations of the study include a small sample size, and that all the students were administered objective and subjective examination in the same order, which could be randomized in future studies. Further, a subjective reporting questionnaire can be used to assess perceived fatigue in the subjects.

## Conclusions

The present study demonstrated that three hours of continuous writing during subjective (essay-type) and objective (MCQ-type) examinations did not produce any significant alteration in the MNCV of the median nerve in the dominant hand of first-year medical students. Despite the absence of examination-related effects, a consistent and significant difference in NCV was observed between the male and female participants. This finding highlights the potential influence of biological or physiological sex-related factors on nerve conduction characteristics and warrants further detailed investigation. Given the study’s small sample size and fixed sequence of examinations, future research with a larger sample size and a randomized exam order is required to enhance generalizability. Additionally, incorporating subjective fatigue assessment tools and exploring variables such as handedness may provide deeper insights into individual differences in NCV. Such studies could help determine whether prolonged academic tasks contribute subtly to neuromuscular fatigue or performance changes not detected in the present study.
